# Liver transplantation for gastroenteropancreatic neuroendocrine liver metastasis: optimal patient selection and perioperative management in the era of multimodal treatments

**DOI:** 10.1007/s00535-024-02166-z

**Published:** 2024-11-15

**Authors:** Yosuke Kasai, Takashi Ito, Toshihiko Masui, Kazuyuki Nagai, Takayuki Anazawa, Yoichiro Uchida, Takamichi Ishii, Koji Umeshita, Susumu Eguchi, Yuji Soejima, Hideki Ohdan, Etsuro Hatano

**Affiliations:** 1 Department of Surgery, Graduate School of Medicine, Kyoto University, 54 Kawahara-cho, Shogoin, Sakyo-ku, Kyoto, 606-8507 Japan; 2 Department of Surgery, Kurashiki Central Hospital, Okayama, Japan; 3 Department of Gastroenterological Surgery, Graduate School of Medicine, Osaka University, Suita, Japan; 4 Osaka International Cancer Institute, Osaka, Japan; 5 Department of Surgery, Graduate School of Biomedical Sciences, Nagasaki University, Nagasaki, Japan; 6 Department of Surgery, Shinshu University School of Medicine, Matsumoto, Japan; 7 Department of Gastroenterological and Transplant Surgery Graduate School of Biomedical and Health Sciences, Hiroshima University, Hiroshima, Japan

**Keywords:** Neuroendocrine liver metastasis, Living donor liver transplantation, Multimodal treatment, Peptide receptor radionuclide therapy

## Abstract

Gastroenteropancreatic neuroendocrine tumors (NET) often metastasize to the liver. Although curative liver resection provides a favorable prognosis for patients with neuroendocrine liver metastasis (NELM), with a 5-year survival rate of 70–80%, recurrence is almost inevitable, mainly in the remnant liver. In Western countries, liver transplantation (LT) has been performed in patients with NELM, with the objective of complete removal of macro- and micro-NELMs. However, prognosis had been unsatisfactory, with 5-year overall survival and recurrence-free survival rates of approximately 50 and 30%, respectively. In 2007, the Milan criteria were proposed as indications for LT for NELM. The criteria included: (1) confirmed histology of NET-G1 or G2; (2) a primary tumor drained by the portal system and all extrahepatic diseases removed with curative resection before LT; (3) liver involvement ≤50%; (4) good response or stable disease for at least 6 months before LT; (5) age ≤ 55 years. A subsequent report demonstrated outstanding LT outcomes for NELM within the Milan criteria, with 5-year overall survival and recurrence rates of 97 and 13%, respectively. In Japan, living donor LT (LDLT) for NELM has been performed sporadically in only 16 patients by 2021 in Japan; however, no consensus has been reached on the indications or perioperative management of LDLT. This article presents the outcomes of these 16 patients who underwent LDLT in Japan and reviews the literature to clarify optimal indications and perioperative management of LDLT for NELM in the era of novel multimodal treatments.

## Introduction

Neuroendocrine neoplasms (NENs) are relatively rare, but their incidence has increased over the past few decades [1]. In Japan, the age-adjusted annual incidence of gastroenteropancreatic (GEP)-NEN was 3.53 per 100,000 population in 2016 [2]. The liver is the most common site of metastasis from GEP-NEN [3]. In analyses of the Surveillance, Epidemiology and End Results database, 36 and 12% of patients with pancreatic and gastrointestinal NENs, respectively, had synchronous neuroendocrine liver metastasis (NELM) and 86% of those had liver-only metastases [4, 5]. The dominance of liver metastasis is due to the anatomical factor that the primary sites are drained by portal systems and the possible biological factor of the high affinity between NEN and the liver microenvironment.

Numerous retrospective studies have shown a favorable prognosis for curative or even cytoreductive liver resection (LR) for NELM, with a 5-year overall survival (OS) rate of 70–80% [6–9]. Consequently, several guidelines have recommended LR for NELM [10–14]. A multi-institutional Japanese retrospective study, including 222 surgical cases of NELM, reported a 5-year OS rate of 70% [15]. In contrast, the 5-year recurrence-free survival (RFS) rate after R0/1 resection was only 13%. More than 90% of recurrences occurred in the remnant liver. These findings suggest that micrometastases are already present in the remnant liver at the time of LR and that partial LR cannot provide a genuine cure for patients with NELM.

In Western countries, liver transplantation (LT) has been performed in patients with NELM, with the theoretical aim of complete removal of macro- and micro-NELMs. However, as shown in Table 1 [16–24], the prognosis in the previous era was unsatisfactory, with 5-year OS and RFS rates of approximately 50 and 30%, respectively. Since immunosuppressive therapy can cause tumor progression, which can result in a poor prognosis, tumors with a high malignant potential should be a contraindication to LT. Based on previous findings, Mazzaferro et al. proposed the Milan criteria to define indications for LT in patients with NELM. Briefly, the Milan criteria were as follows: (1) confirmed histology of neuroendocrine tumor (NET) -G1 or G2; (2) primary tumor drained by the portal system and all extrahepatic diseases removed with curative resection before LT; (3) liver involvement ≤50%; (4) good response or stable disease for at least 6 months before LT; (5) age ≤ 55 years [25]. Their subsequent report demonstrated outstanding outcomes following LT for NELM within the Milan criteria, with 5-year OS and recurrence rates of 97 and 13%, respectively [26]. Under such strict selection criteria, LT with perioperative medical management using evolving multimodal treatments could enhance the chances of cure for patients with NELM.

**Table 1:** List of original articles including ≥50 patients who underwent LT for NELM

Author	Year	Study period	#LT patients	5-year RFS (%)	5-year OS (%)	Remarks
Le Treut [16]	2008	1989–2005	85	20	47	Multicenter study in France
Gedaly [17]	2011	1988–2008	150	NA	49	UNOS database, comparing with LT for hepatocellular carcinoma
Nguyen [18]	2011	1988–2011	184	NA	49	UNOS/OPTN database
Le Treut [19]	2013	1982–2009	213	30	52	Multicenter study in Europe
Sher [20]	2015	1988–2012	85	NA	52	Multicenter study
Nobel [21]	2015	2002–2014	128	NA	63	UNOS database
Houben [22]	2021	1988–2017	100	NA	56	Collaborative transplant study database
Valvi [23]	2021	1988–2018	206	44	65	UNOS database
Eshmuminov [24]	2023	1988–2021	225	62	74	Multicenter study, comparing with liver resection

*LT* liver transplantation, *NA* not available, *NELM* neuroendocrine liver metastasis, *OPTN* organ procurement and transplantation network, *UNOS* united network for organ sharing

In Japan, living donor LT (LDLT) for NELM has been performed sporadically in only 16 patients by the end of 2021, according to the registry of the Japanese Liver Transplantation Society (JLTS) [27]. However, no consensus has been reached regarding the indications or perioperative treatment of LDLT. Herein, we present the outcomes of these 16 patients who underwent LDLT in Japan and review the literature to clarify the optimal indications and perioperative management of LDLT for NELM in the era of novel multimodal treatments.

## Outcome of 16 patients with LDLT in Japan

Data were extracted from the JLTS registry database [27]. The registry protocol was comprehensively approved by the institutional review board of the National Center for Global Health and Medicine (approval number: NCGM-S-004559-00), and the use of the registry data for publication was approved by the registry committee of JLTS. The requirement for written informed consent was waived due to the de-identified nature of the database.

**Fig. 1:**
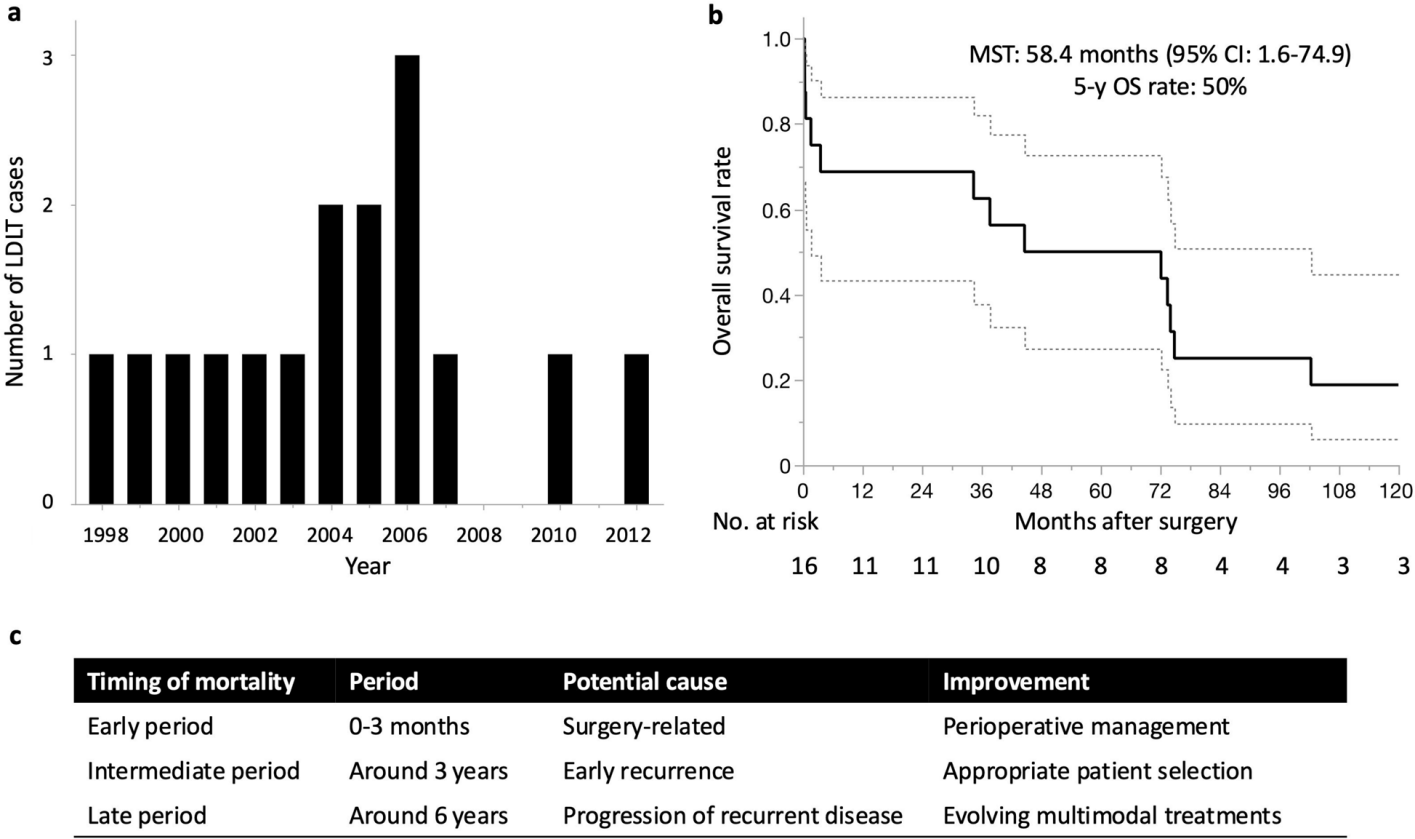
LDLT for NELM in Japan. **a** Annual number of LDLT cases. **b** Kaplan–Meier OS curve after LDLT for NELM. **c** Timing of mortality, their potential cause, and their improvement. *CI* confidence interval, *LDLT* living donor liver transplantation, *MST* median survival time, *NELM* neuroendocrine liver metastasis, *OS* overall survival

A total of 16 patients were identified by the end of 2021. Fourteen of the 16 patients (88%) underwent LDLT in or before 2007 (Fig. 1a), when the Milan criteria were proposed. No patients underwent LDLT for NELM after 2012 in Japan. The demographics of the patients are summarized in Table 2. The median age was 30.5 and all patients were under 60 years of age. Primary tumors were located in the pancreas in 12 patients (75%), in the rectum in 3 patients (19%), and in the stomach in 1 patient (6%). Most of the patients had non-functional or unspecified tumors (9 patients, 56%), while the others had functional tumors, including gastrinoma and insulinoma in 2 patient each. Four patients (25%) died within 90 days of LDLT. The median survival time of 16 patients was 58.4 months [95% confidence interval (CI): 1.6–74.9] and the 5-year OS rate was 50% (Fig. 1b).

**Table 2:** Demographics of 16 patients who underwent LDLT for NELM in Japan

Variables	Median (range) or *N* (%)
Age	30.5 (23–59)
Sex M/F	8 (50%)/8 (50%)
Primary origin	
Pancreas	12 (75%)
Rectum	3 (19%)
Stomach	1 (6%)
Function	
Nonfunctional or unspecified	9 (56%)
Gastrinoma	2 (13%)
Insulinoma	2 (13%)
ACTHoma	1 (6%)
Somatostatinoma	1 (6%)
PPoma	1 (6%)
Graft type	
Right lobe	8 (50%)
Left lobe	8 (50%)
ABO compatibility	
Identical	13 (81%)
Compatible	3 (19%)
30-day mortality	3 (19%)
90-day mortality	4 (25%)

*ACTHoma* adrenocorticotropic hormone-producing tumor, *LDLT* living donor liver transplantation, *NELM* neuroendocrine liver metastasis, *PPoma* polypeptide-secreting tumor

The prognostic outcome of the 16 patients were comparable to that reported in previous cases from Western countries in earlier eras (Table 1). Although the discouraging outcome in the previous era would have impaired the promotion of LDLT for patients with NELM in Japan afterward, we could find some improvements through analyzing the registry data. In Fig. 1b, we found 3 timepoints of survival curve drop: (1) early period (0–3 months after LT); (2) intermediate period (around 3 years after LT); and (3) late period (around 6 years after LT). The cause of mortality during each period might be surgery-related for the early period, early recurrence for the intermediate period, and progression of recurrent disease for the late period (Fig. 1c), though we do not have detailed data regarding recurrence or clinical course after recurrence. Surgery-related mortality have been overcome through improved perioperative management. Actually, the risk of mortality after LDLT for adult patients has decreased to less than half in the period of 2011–2021 compared to 1989–1999 and 2000–2010 in Japan [28]. Early recurrence could be excluded through appropriate patient selection. In the JLTS registry data, 14 of the 16 patients were the cases before the era of the Milan criteria, and might not have met the optimal indication criteria of LT in the current era. Time to progression of the recurrent disease could be prolonged through evolving multimodal treatments for NEN. Collectively, it is highly expected that the prognostic outcome after LDLT for NELM in the current era would substantially exceed that of the registry data. This review article aimed to verify these points.

## Comparison of long-term outcomes between LT and non-LT treatments

No randomized controlled trials (RCTs) have compared the long-term outcomes between LT and LR or medical treatments. A recent international multi-institutional retrospective study by Eshmuminov et al. enrolled 225 and 230 patients who underwent LT and LR for NELM, respectively, and compared long-term outcomes using propensity score matching [24]. After the propensity score matching, both progression-free survival (PFS) and OS were significantly better in the LT group than those in the LR group (5-year PFS rates of 64 and 14%, respectively, with 5-year OS rates of 75 and 68%, respectively). However, in these comparisons after the propensity score matching, the number of lesions was markedly unbalanced between the groups, with median numbers (interquartile range) for the LT and LR groups of 10 (7–100) and 1 (1–3), respectively. In contrast, the proportion of patients with stable disease for at least 6 months preoperatively, representing the indolent behavior of the tumor, might be lower in the LR group than in the LT group (data not shown) because preoperative treatment before LR has not been practical. The latter bias was addressed through a subgroup analysis of patients within the Milan criteria, where the OS benefit of LT over LR was further enhanced. These biases indicate that LT was performed generally in patients with unresectable NELM and that the indications hardly overlapped between LT and LR.

Maspero et al. compared the long-term outcomes of LT and LR for NELM within the Milan criteria [29]. They specified that LR was offered to patients with resectable disease, whereas those with unresectable NELM were considered for LT. Therefore, the characteristics definitely differed between the LT and LR groups (including the proportion of patients with liver involvement ≥25% of 58% vs. 21%, respectively). However, OS and RFS were significantly better in the LT group than in the LR group (10-year OS rates of 93 and 75%, respectively, and 10-year RFS rates of 52 and 18%, respectively).

Overall, although the comparison between LT and LR in retrospective studies has been considerably biased, the favorable long-term outcomes of LT strongly suggest that LT can be a curative treatment even for unresectable NELM under strict selection criteria.

Mazzaferro et al. compared the long-term outcomes of LT and non-LT treatments for NELM within the Milan criteria [26]. They screened 280 patients with NELM who were referred to their center for LT, and 86 met the Milan criteria: 42 patients underwent LT, whereas the remaining 46 did not due to noncompliance, refusal, or transplant list unavailability. The burden of liver tumors was well balanced between the LT and non-LT groups, while the proportion of G1 was significantly higher in the LT group than in the non-LT group (79 vs. 52%, respectively). The 10-year OS rates were 89 and 22% for the LT and non-LT groups, respectively, and the survival benefit of LT over non-LT at 10-year follow-up was 48.6 months (95% CI: 35.5–61.8). However, it should be noted that the study period was from 1995 to 2010, when most of the current therapeutic modalities were not practically available.

The therapeutic modalities for NEN have evolved drastically over the last decade; somatostatin analogs (SSA) [30, 31], molecular targeted agents (MTA), including everolimus [32, 33] and sunitinib [34], and peptide receptor radionuclide therapy (PRRT) using ^177^Lu-DOTA TATE [35, 36] have achieved prolonged PFS in RCTs. Above all, PRRT was shown to reduce the risk of disease progression or death by 79% in patients with progressive unresectable midgut NET compared to active control in the NETTER-1 trial published in 2017 [35]. A large-scale study by the Erasmus Medical Center also showed favorable short- and long-term results of PRRT, with a response rate of 39% and a median OS of 63 months [37]. Based on these results, the US Food and Drug Administration-approved PRRT using ^177^Lu-DOTA TATE in 2018. In Japan, it has been covered by insurance since 2021. Furthermore, NETTER-2 trial recently demonstrated the effectiveness of PRRT as the first line therapy for GEP-NET-G2/G3 (Ki-67 index of 10–55%) [36]. Multimodal treatments, including these active agents, would certainly improve the prognosis of non-LT patients, and the survival benefit of LT over non-LT treatments, as shown by Mazzaferro et al. [26], might be attenuated in the modern era. Nevertheless, these non-LT treatments do not provide a cure for the disease. The complete response rate to PRRT was only 2% in the Erasmus Medical Center trial [37]. Furthermore, despite the remarkable prolongation of PFS by PRRT compared to active control in the NETTER-1 trial, the final OS analysis of the NETTER-1 trial did not show significant differences between the PRRT and active control groups (hazard ratio of 0.84, 95% CI: 0.60–1.17) and the median OS was only 48.0 months for the PRRT group [38]. Therefore, it is highly anticipated that most young patients would finally die of NEN after a long tumor-bearing period. Thus, LT may be a useful modality toward a cure of diseases in young patients with unresectable NELM under strict selection criteria.

## Indications of LDLT for patients with NELM

As favorable outcomes of LT for NELM within the Milan criteria have been demonstrated, the Milan criteria should be the standard indications for LDLT in Japan. Furthermore, the validity of operating on healthy donors should be ensured. As mentioned above, Eshmuminov et al. showed that LT was superior to LR for OS and PFS in a comparison of propensity score-matched patients, although patient backgrounds were not optimally balanced between the study groups [24]. Nevertheless, from the viewpoint of donor safety, performing LDLT for resectable NELM would not be valid. LR should be prioritized over LDLT for resectable NELM, allowing salvage LT for recurrent NELM after LR. Below, we will describe the details of the Milan criteria.

### (1) Confirmed histology of NET-G1 or G2

**Fig. 2:**
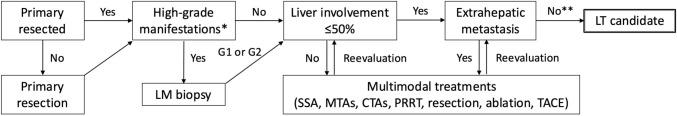
Screening for LT based on the Milan criteria. *High-grade manifestations include Ki-67 index ≥10% of the primary tumor, discrepantly rapid progression of NELM for the Ki-67 index of the primary tumor, decreased avidity of somatostatin receptor imaging, and increased avidity of ^18^F-fludeoxyglucose positron emission tomography. **Extrahepatic metastasis should be ruled out by ^68^Ga-DOTA positron emission tomography. *CTA* cytotoxic agent, *LM* liver metastasis, *LT* liver transplantation, *MTA* molecular targeted agent, *PRRT* peptide receptor radionuclide therapy, *SSA* somatostatin analogue, *TACE* transcatheter arterial chemoembolization

The histological grade represents the malignant potential of NEN. LT should be avoided for tumors with high malignant potential due to the high risk of rapid recurrence under immunosuppression. NET-G3 and poorly differentiated neuroendocrine carcinomas are regarded to be contraindications for LT because of their high malignant potential [19]. The Milan criteria include G2 with a Ki-67 index ranging from 3 to 20%. However, some other LT criteria exclude tumors with Ki-67 index ≥10% [24]. The optimal cutoff point of the Ki-67 index for the selection of LT has yet to be determined. The histological grade is often evaluated by pathological examination of the resected specimen of the primary tumor. A grade discrepancy between primary tumor and liver metastasis was observed in about 30% of cases and most discrepancies were upgrade in liver metastasis [39]. Therefore, a tumor biopsy of representative liver metastases should be considered if there are high-grade manifestations (Fig. 2).

### (2) Primary tumor drained by the portal system and all extrahepatic diseases removed with curative resection prior to LT

The theoretical concept of LT for NELM is complete removal of macro- and micro-NELMs. This concept is effective only for primary sites drained by the portal system, because primary tumors drained by systemic circulation (such as pulmonary/bronchial/thymus NEN) have substantially high risk of extrahepatic metastasis, including lymph nodes, bone, and brain, compared to gastro-entero-pancreatic NEN [3].

Regarding simultaneous resection of the primary tumor with LT, the addition of a “major procedure,” including pancreaticoduodenectomy or all types of pancreatectomy and multivisceral transplantation, is considered a poor prognostic factor [19, 20, 40]. Furthermore, concomitant gastrointestinal procedures with moderate risk of infection should be avoided for LT. Although the prognostic importance of primary tumor resection in patients with synchronous unresectable metastases has been poorly defined [11, 12], resection of the primary tumor with regional lymph node dissection might be considered for the purpose of staging and grading for candidates of LT.

Since most recurrence after LT occurs at extrahepatic sites [29, 41], the presence of extrahepatic metastases that were undetectable on conventional contrast-enhanced computed tomography or magnetic resonance imaging could be the cause of recurrence after LT. Therefore, best efforts should be made to rule out such occult metastases. Somatostatin receptor scintigraphy (Octreoscan®) is used for the diagnosis of NEN, but its detectability of small lesions (<1 cm) is low. To exclude extrahepatic metastases for the selection of LT, ^68^ Ga DOTA TOC (or DOTA TATE)-positron emission tomography is preferable to somatostatin receptor scintigraphy because of its high detectability [42, 43], although it has not been covered by insurance in Japan as of today. In cases of resected extrahepatic metastases, the absence of new extrahepatic lesions for >6 months afterward is a requirement for LT.

### (3) Liver involvement ≤ 50%

Liver involvement is a representative index of NELM tumor burden and has been generally used in NEN studies [30, 31, 33, 44]. However, most studies did not define detailed methods for calculating liver involvement and some might have been determined by subjective estimation. In Japan, most hepatobiliary surgeons use volume analysis software such as SYNAPSE VINCENT (Fujifilm Medical Co., Ltd., Tokyo, Japan) [45]. This facilitates the computation of the volume of the normal liver parenchyma, total tumors, and liver involvement [46]. Such objective measurements are mandatory to further elucidate the prognostic impact of tumor burden on LT.

Items (1)–(3) can be used to screen for patients eligible for LT (Fig. 2).

### (4) Good response or stable disease for at least 6 months prior to LT

**Fig. 3:**
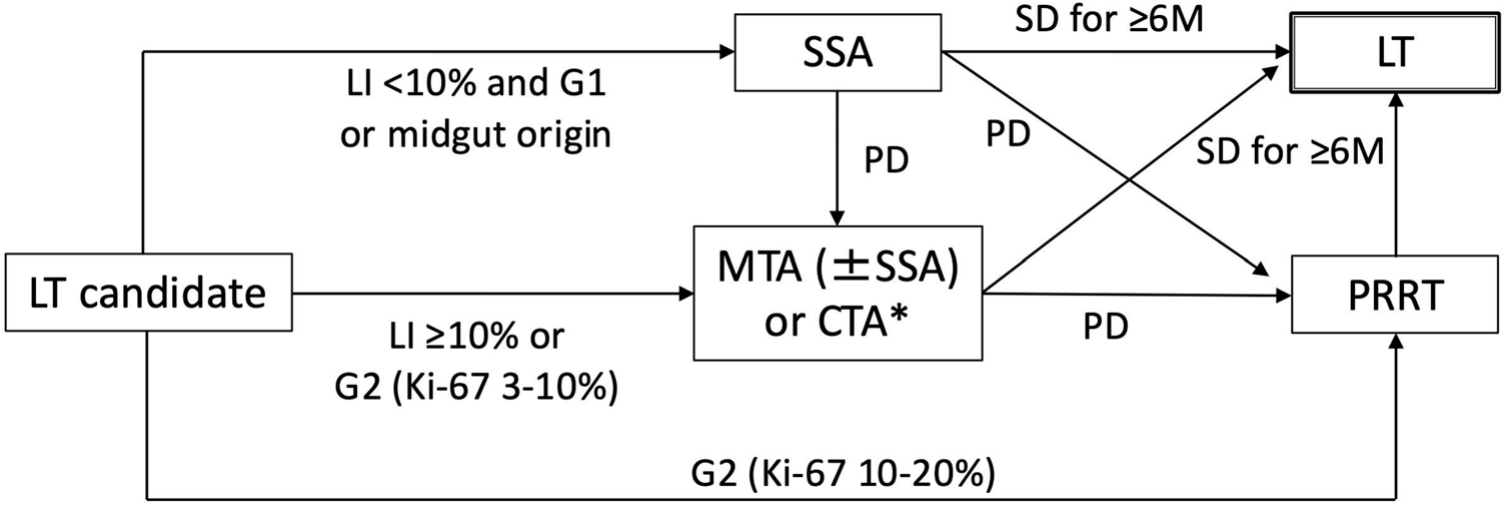
Bridging therapy to LT. *CTA is indicated only for pancreatic neuroendocrine neoplasm. *CTA* cytotoxic agent, *LI* liver involvement, *LT* liver transplantation, *MTA* molecular targeted agent, *PD* progressive disease, *PRRT* peptide receptor radionuclide therapy, *SD* stable disease, *SSA* somatostatin analogue

Disease control for >6 months is required to ascertain the malignant potential, response to medical treatments, and the presence or absence of emerging extrahepatic metastasis. As mentioned above, medical treatments for NEN have evolved drastically since 2007, when the Milan criteria were proposed. Ikeda et al. proposed a treatment selection map for metastatic pancreatic NEN based on the Ki-67 index and liver involvement as follows [47]: SSA treatment for patients with a relatively small tumor burden and low Ki-67 index [31], cytotoxic agents for patients with a relatively large tumor burden and high Ki-67 index [48, 49], and MTAs for patients with intermediate tumor volume and/or Ki-67 index [32, 34]. For gastrointestinal, non-midgut NEN, SSA and everolimus can be used as for pancreatic NEN, while the SSA coverage can be expanded to tumors with a larger tumor burden or higher Ki-67 index for midgut NEN due to its indolent behavior [50]. Referring to these treatment options, medical treatments may be used as the bridging therapies to LT, as shown in Fig. 3. PRRT can be offered to patients with progressive disease after SSA, MTA, or cytotoxic agents, or as the first-line therapy for those with Ki-67 index ≥10%, and subsequent LT should be planned while the disease is stably controlled by PRRT.

In Western countries, where deceased donor LT (DDLT) is the mainstay, candidates do not always receive LT on demand, even if their diseases are well controlled by medical treatments. Mazzaferro et al. showed that the median waiting time from board decision to LT was 6.8 months (interquartile range 1.7–8.1) [26]. A long waiting time could lead to tumor progression, preventing candidates from receiving LT. Conversely, LDLT has an advantage over DDLT in terms of the schedule of medical treatments and response evaluation, and LT can be determined actively.

### (5) Age ≤ 55 years

The Milan criteria indicate age as relative criteria and accept LT for patients aged >55 years. Nevertheless, Mazzaferro et al. showed that patients aged ≥55 had significantly higher risk of mortality than middle-aged patients (42–54, hazard ratio of 3.72) [26]. For patients aged >55 years, the indications for LT should be individually determined based on the availability of other therapeutic options, the control of hormonal symptoms, and comorbidities.

To our knowledge, there have been no relevant studies focusing on LT for NELM associated with hereditary syndrome, including multiple endocrine neoplasia type 1 (MEN-1) and von Hippel–Lindau (VHL) disease. MEN-1-associated NEN is characterized by multiplicity and functional tumors. In particular, Zollinger–Ellison syndrome caused by gastrinoma, mainly in the duodenum, is present in approximately 30% of patients with MEN-1 [51]. From the viewpoint of hormonal symptom control, it would be acceptable to perform LT for patients with MEN-1 associated NELM within Milan criteria [51]. However, residual tumors in the duodenum or pancreas should be carefully ruled out because of its potential multiplicity. VHL disease-associated pancreatic NENs are also characterized by multiplicity, but most of them are non-functional [52, 53]. Approximately, 50% of patients with VHL disease are associated with hemangioblastoma of the central nervous system and renal cell carcinoma, respectively, and these malignancies are considered to prognosticate patients with VHL disease [53, 54]. Therefore, VHL disease-associated NELM would not be suited for LT even if the Milan criteria were met. In addition, for patients with hereditary NEN, their relatives may also possess the same hereditary disease and would not be eligible for living donor.

## Immunosuppressive therapy after LT

Tacrolimus, a calcineurin inhibitor, is generally used as an immunosuppressant following LT. Everolimus or sirolimus, inhibitors of the mammalian target of rapamycin (mTOR), is another option of immunosuppressant. Recently, a meta-analysis showed that sirolimus or everolimus was associated with a reduced risk of recurrence or death in patients who underwent LT for hepatocellular carcinoma [55]. Since mTOR signaling is one of the key pathways in NEN [56] and everolimus has been shown to reduce the risk of progression or death in unresectable progressive NEN [32, 33], everolimus can be expected to have a preventive effect against recurrence as well as immunosuppression. However, the standard therapeutic dose of everolimus differs across indications (10 mg daily for NEN and 1 mg twice daily as an immunosuppressant after LT). In addition, no study (including retrospective studies) has examined the preventive effects against recurrence after LT for NELM, and more evidence is needed on this issue.

## Managements after recurrence

Under immunosuppression after LT for malignancy, rapid progression occurs frequently once recurrence occurs. We previously showed that 23 of the 164 patients who underwent LDLT for hepatocellular carcinoma experienced recurrence and the median survival time after recurrence was only 15 months [57]. Therefore, selection criteria are required that exclude tumors with high malignant potential [58]. However, recurrence occurs to some extent and appropriate management is required for recurrent disease.

Sposito et al. reported the results of 32 patients who experienced recurrence after LT for NELM (including 25 patients meeting the Milan criteria) [41]. The median time between LT and recurrence was 82.9 months. The most frequent site of recurrence was the abdominal lymph nodes (19 patients, 59%), followed by the peritoneum and lung (2 patients, 6% each). Notably, intrahepatic recurrence was observed in only one patient (3%). The authors aggressively treated patients with recurrence using surgery (14 patients, 44%), PRRT (2 patients, 6%), SSA (11 patients, 34%), or chemotherapy (3 patients, 9%). As a result, survival after recurrence was favorable, with a 5-year survival rate after recurrence of 76%. These findings indicate that appropriate patient selection for LT can achieve good disease control and long-term survival through multimodal treatment even after recurrence.

## Conclusions

Herein, we describe optimal indications and perioperative management of LDLT for NELM. The Milan criteria would be the standard indication for LDLT in Japan: GEP-NET-G1 or G2, liver-limited disease, liver involvement ≤50%, stable disease for ≥6 months, and age ≤55 years. Although the outcome of LDLT for NELM in the previous era in Japan was unsatisfactory, we verified that the outcome would improve substantially in the current era through decreased risk of surgery-related mortality, strict patient selection criteria, and evolving multimodal treatments including PRRT. Whereas PRRT would not provide a cure despite its overwhelming antitumor effects, LT based on such a rigorous strategy could enhance the chances of curing intractable NELM.
